# Supplementary material for: Molecular Dynamics Study of Clathrate-like Ordering of Water in Supersaturated Methane Solution at Low Pressure
Source: Molecules. 2023 Mar 26;28(7):2960. doi: 10.3390/molecules28072960 (PMC10095827; doi:10.3390/molecules28072960)
Supplement: Supplementary file 1 [file molecules-28-02960-s001.zip › Supplementary_Molecules_3.pdf]

Molecular dynamics study of clathrate-like ordering of water in supersaturated methane solution at low pressure

Rodion V. Belosludov [1,*], Kirill V. Gets [2,3], Ravil K. Zhdanov [2,3], Yulia Yu. Bozhko [2,3], Vladimir R. Belosludov [2,3], Li-Jen Chen [4] and Yoshiyuki Kawazoe [5,6,7]

[1] Institute for Materials Research, Tohoku University, 980-8577 Sendai, Japan;
[2] *Nikolaev Institute of Inorganic Chemistry, Siberian Branch, Russian Academy of Sciences, Novosibirsk, 630090 Russia;*
[3] *Novosibirsk State University, Novosibirsk, 630090 Russia;*
[4] *Department of Chemical Engineering, National Taiwan University, 10617 Taipei, Taiwan;*
[5] *New Industry Creation Hatchery Center, Tohoku University, 980-8579, Sendai, Japan;*
[6] *Department of Physics and Nanotechnology, SRM Institute of Science and Technology, 603203, Kattankurathur, Tamil Nadu, India;*
[7] *School of Physics, Institute of Science, Suranaree University of Technology, 30000, Nakhon Ratchasima, Thailand;*

* *Correspondence: belosludov.rodion.vladimirovich.a3@tohoku.ac.jp;*

1. Barostat and thermostat time parameters choice.

In MD simulations of sufficiently large systems, 100/1000 dumping constants is a universal choice due to the sufficient number of modeling particles that reduce the dispersion of thermodynamic parameters. For small systems, such selection of dumping parameters may be not enough, which can be leveled by increasing the time constants at the cost of a slight increase in the temperature and pressure reaching the equilibrium state, which is not critical in our case. In theoretical works, different relaxation times of the thermostat and barostat are used. Low 100/1000 [S1, S2], 200/500 [S3], 100/500 [S4] and very high 2000/4000 [S4], 1000/4000 [S5,S6], 1000/5000 [S7], 1000/2500 [S8].

2. Pre-nucleation: spatial distribution of cavities

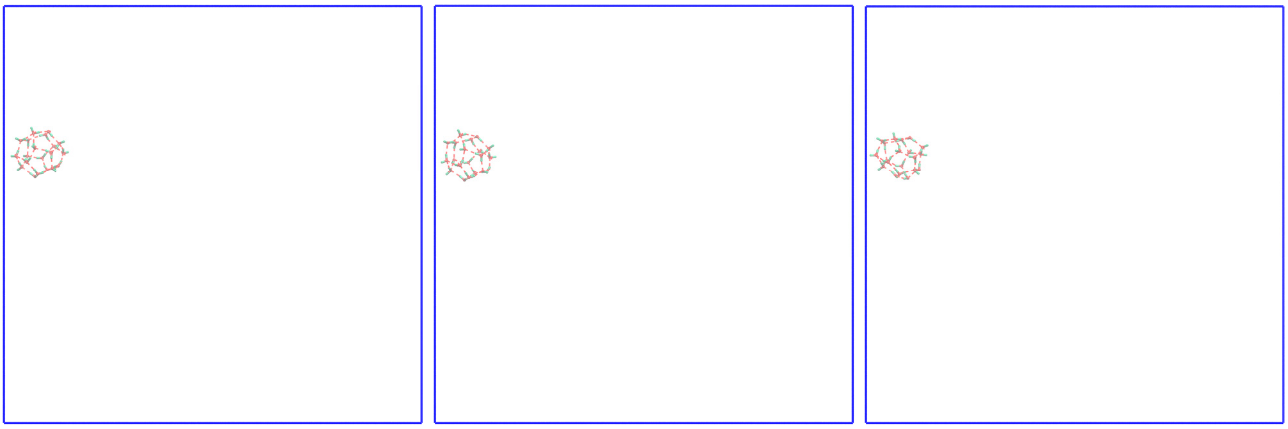

t = 1500 ps t = 1512 ps t = 1558 ps

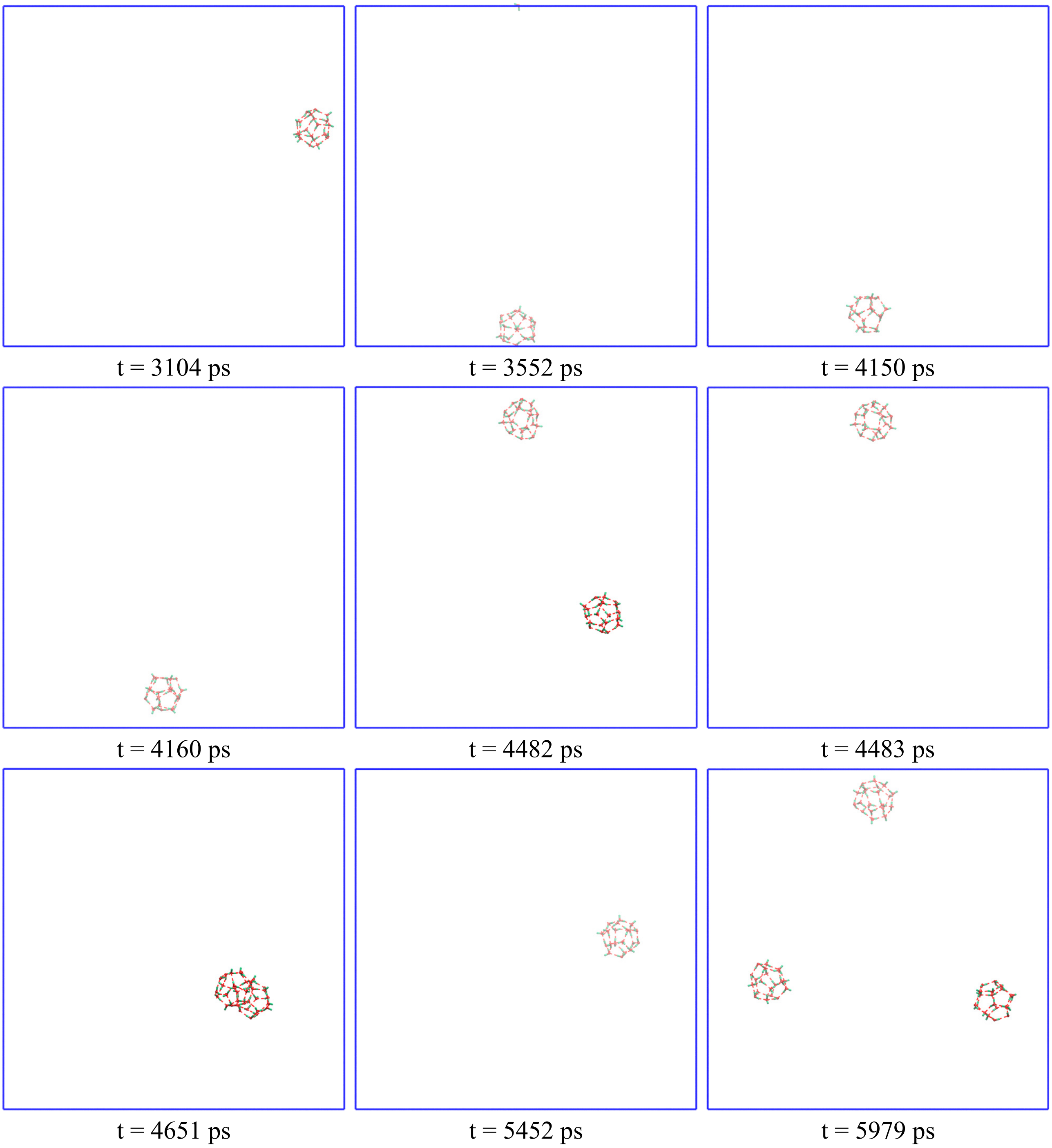
t = 3104 ps
t = 3552 ps
t = 4150 ps
t = 4160 ps
t = 4482 ps
t = 4483 ps
t = 4651 ps
t = 5452 ps
t = 5979 ps

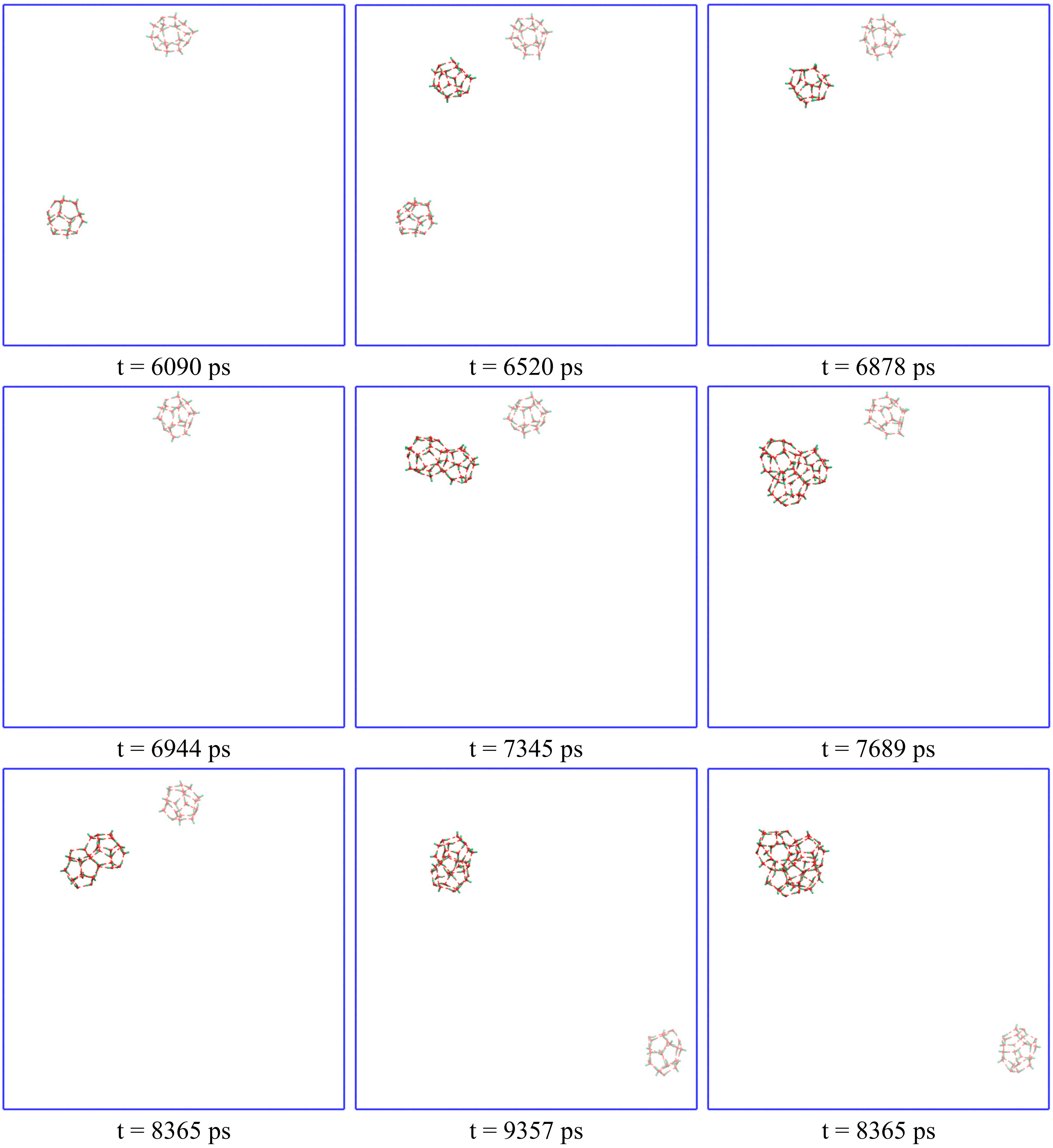

Figure **S1**. Spatial distribution of small and large cavities.

3. <u>Pre-nucleation: gas-oxygen RDF</u>

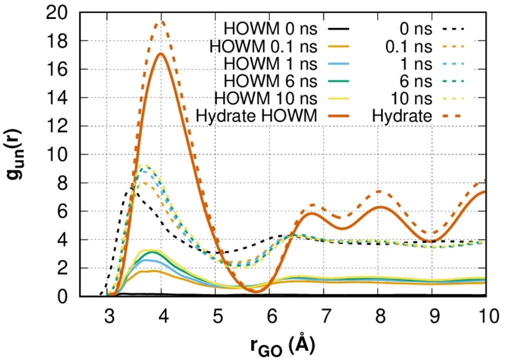

Figure S2. Unnormed by number of G-O pairs methane-oxygen (eigen $F_3 < 0.025$) RDF of 0.6 methane solution with 0.01 Å step in comparison to bulk hydrate data at t = 0.5 ns under the same conditions. *Dotted lines* show the dependence for all water molecules, *solid lines* – for HOWMs.

Figure S2 shows the density unnormalized methane–oxygen RDF for HOWMs and all water molecules for solution and bulk hydrate. Values of this distribution grow proportionally to the number of HOWMs. Growth of peak with a maximum of 4 Å indicates an increase in the number of water molecules surrounding methane molecules both for HOW sub-system and for whole H-bond network. However the most significant relative growth is observed for HOWMs (2 times). Whole network undergoes a change of the order of 15-20 % that corresponds to the ratio of HOWMs. An increase indicates a gradual transition from an amorphous environment to a crystalline one. The main change in the structure of the solution and HOW sub-system takes place before the start of stable hydrate formation.

4. <u>Time dependence of system pressure and temperature</u>

Methane concentrations in aqueous solution is following: 0.2 – 3.36 mol%, 0.4 – 6.5 mol%, 0.6 – 9.45 mol%, 0.8 – 12.2 mol%, 1.0 – 14.8 mol%.

The increase in scatter at the end of the smoothed graphs appears due to the peculiarity of Bezier smoothing realized in GnuPlot.

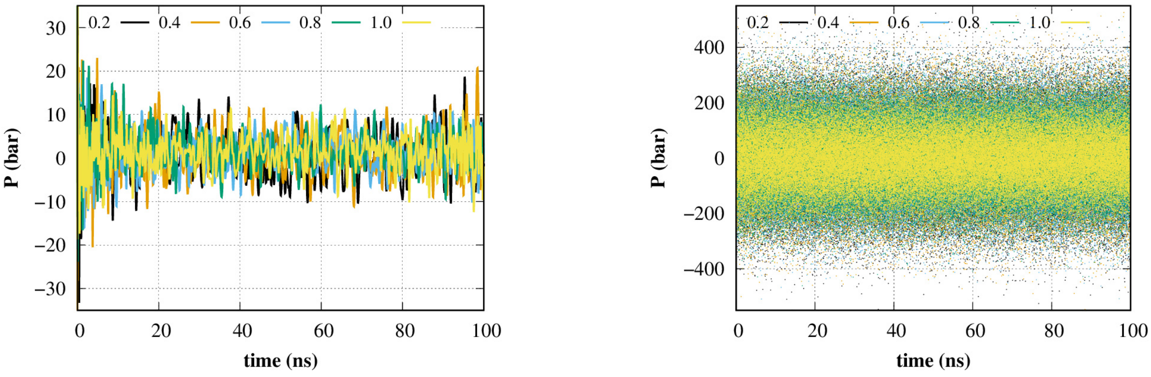

(a) **(b)**

Figure S3. Time dependence of system pressure (*P*): (*a*) smoothed and (*b*) raw data at different methane concentrations.

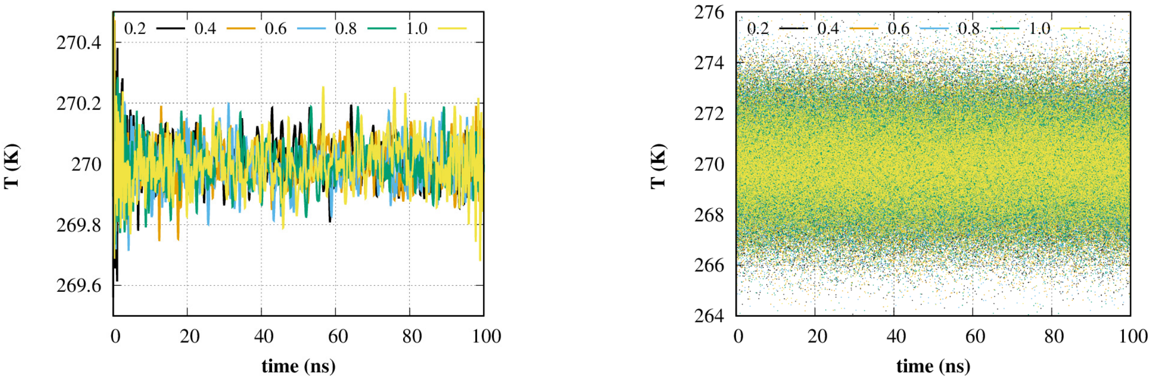

(a) **(b)**

Figure S4. Time dependence of system temperature (T): (a) smoothed and (b) raw data at different methane concentrations.

5. <u>Methane hydrate growth visualization</u>

Supplementary video S1. Evolution of methane+water solution at concentration of 0.2 (3.36 mol%) during 100 ns of modeling. Positions of water molecules, methane molecules and H-bond are smoothed over 1 ns for clearance.

Supplementary video S2. Evolution of methane+water solution at concentration of 0.4 (6.5 mol%) during 100 ns of modeling. Positions of water molecules, methane molecules and H-bond are smoothed over 1 ns for clearance.

Supplementary video S3. Evolution of methane+water solution at concentration of 0.6 (9.45 mol%) during 100 ns of modeling. Positions of water molecules, methane molecules and H-bond are smoothed over 1 ns for clearance.

Supplementary video S4. Evolution of methane+water solution at concentration of 0.8 (12.2 mol%) during 100 ns of modeling. Positions of water molecules, methane molecules and H-bond are smoothed over 1 ns for clearance.

Supplementary video S5. Evolution of methane+water solution at concentration of 1.0 (14.8 mol%) during 100 ns of modeling. Positions of water molecules, methane molecules and H-bond are smoothed over 1 ns for clearance.

6. <u>Number of cavities in other simulation series</u>.

Table S1. Designation (C) of methane (N_M) + water (N_W) solution compositions for large series A systems and composition of smaller systems.

C (series A)	N_M	N_W
0.2	348	10001
0.4	696	10001
0.6	1043	10001
0.8	1391	10001
1.0	1739	10001

	N_M	N_W
Run 1	313	3000
Run 2	313	3000
Run 3	313	3000
Run 4	313	3000

Series A structures contain the same numbers of water and methane molecules as it showed in **Table S1**. Smaller models contain 3000 water molecules and 313 methane molecules (methane concentration as in C = 0.6). Every model has different volume and initial speed distribution of molecules. It showed coinciding results. Difference of sizes may not lead to any significant thermodynamic discrepancy [S9].

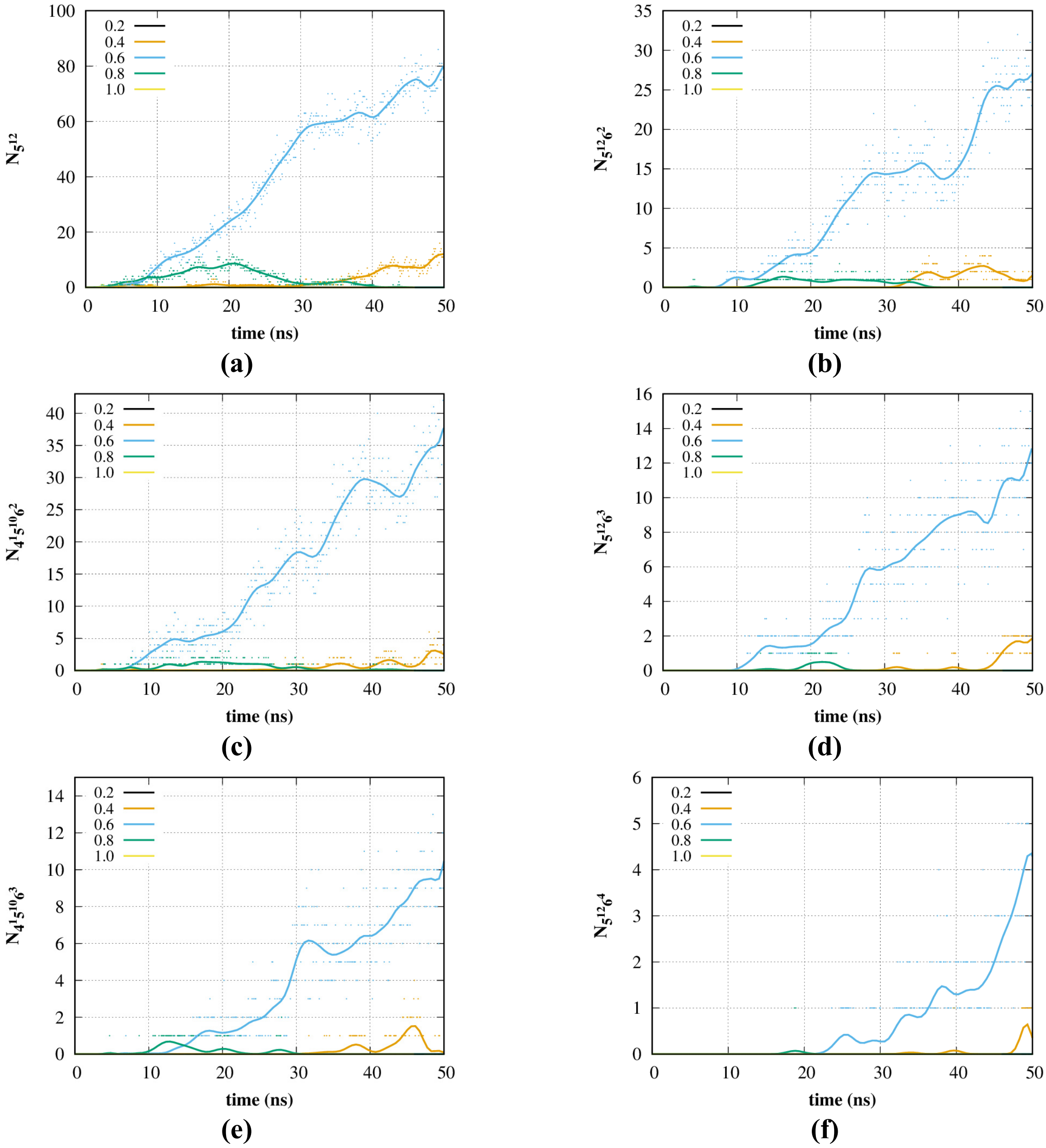

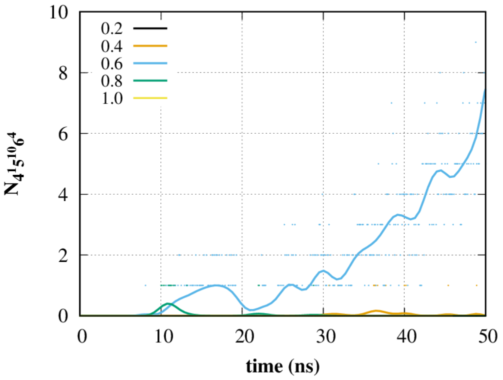

(g)

Figure S5. Time dependence of number of (*a*) 5^{12}, (*b*) $5^{12}6^2$, (*c*) $4^15^{10}6^2$, (*d*) $5^{12}6^3$, (*e*) $4^15^{10}6^3$, (*f*) $5^{12}6^4$, (*g*) $4^15^{10}6^4$ type cavities in series A simulations. *Solid lines* present the smoothed data.

Presented data on the number of hydrate and topologically similar cavities for 0.4 and 0.6 concentrations show the same behavior as for presented in main text series B. Concentrations 0.2 and 1.0 also reveal no hydrate formation like for series B. The main difference for the presented time period is for 0.8 concentration: unstable growth and fast hydrate dissociation in supersaturated solution are in agreement with theoretical results of work [S4], which predicts that the water and gas phase separation occurs for the cases of methane molar concentration is higher than 9.8 mol%.

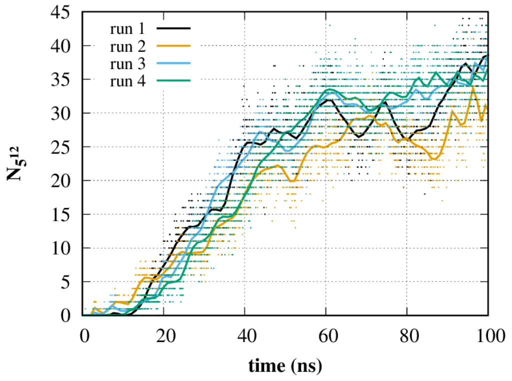

(a)

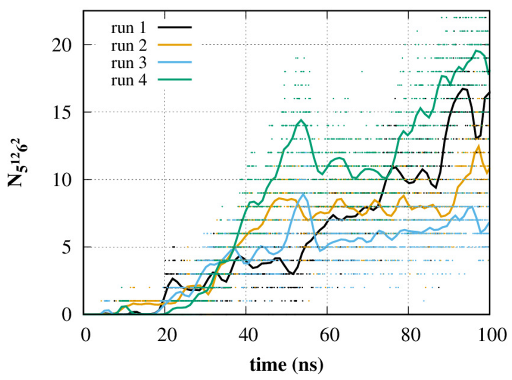

(b)

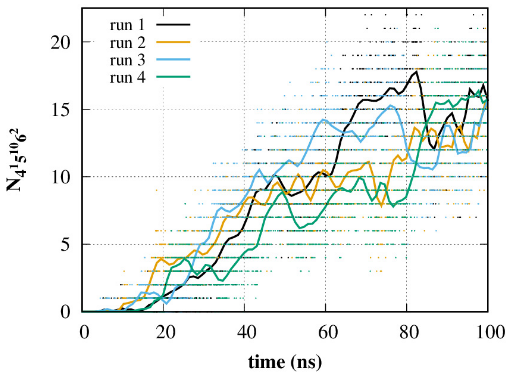

(c)

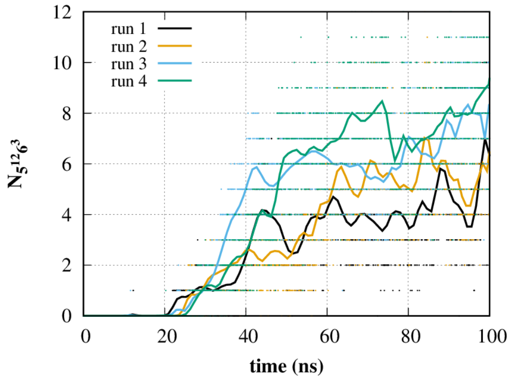

(d)

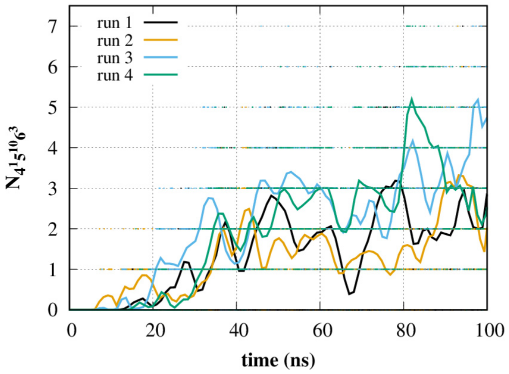

(e)

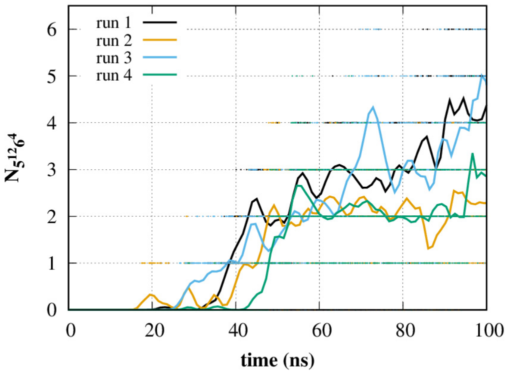

(f)

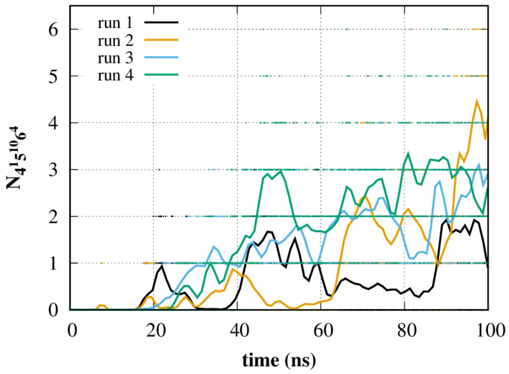

(g)

Figure S6. Time dependence of number of (*a*) 5^{12}, (*b*) $5^{12}6^2$, (*c*) $4^15^{10}6^2$, (*d*) $5^{12}6^3$, (*e*) $4^15^{10}6^3$, (*f*) $5^{12}6^4$, (*g*) $4^15^{10}6^4$ type cavities in smaller models. *Solid lines* present the smoothed data.

References:

[S1] Mi, F., He, Z., Fang, B., Ning, F., & Jiang, G. Molecular insights into the effects of surface property and pore size of non-swelling clay on methane hydrate formation. *Fuel* **311**, 122607 (2022).

[S2] Tung, Y. T., Chen, L. J., Chen, Y. P., & Lin, S. T. Molecular dynamics study on the growth of structure I methane hydrate in aqueous solution of sodium chloride. *J. Phys. Chem. B* **116**, 14115-14125 (2012).

[S3] Bagherzadeh, S. A., Alavi, S., Ripmeester, J., & Englezos, P. Formation of methane nano-bubbles during hydrate decomposition and their effect on hydrate growth. *J. Chem. Phys.* **142**, 214701 (2015).

[S4] Jiménez-Ángeles, F., & Firoozabadi, A. Nucleation of methane hydrates at moderate subcooling by molecular dynamics simulations. *J. Phys. Chem. C* **118**, 11310-11318 (2014).

[S5] Arjun, Berendsen, T. A., & Bolhuis, P. G. Unbiased atomistic insight in the competing nucleation mechanisms of methane hydrates. *PNAS* **116**, 19305-19310 (2019).

[S6] Choudhary, N., Kushwaha, O. S., Bhattacharjee, G., Chakrabarty, S., & Kumar, R. Macro and Molecular Level Insights on Gas Hydrate Growth in the Presence of Hofmeister Salts. *Ind. Eng. Chem. Res.* **59**, 20591-20600 (2020).

[S7] DeFever, R. S., & Sarupria, S. Nucleation mechanism of clathrate hydrates of water-soluble guest molecules. *J. Chem. Phys.* **147**, 204503 (2017).

[S8] English, N. J., John, S. T., & Carey, D. J. Mechanisms for thermal conduction in various polymorphs of methane hydrate. *Phys. Rev. B* **80**, 134306 (2009).

[S9] Conde, M. M., Rovere, M. & Gallo, P. High precision determination of the melting points of water TIP4P/2005 and water TIP4P/Ice models by the direct coexistence technique. *J. Chem. Phys.* **147,** 244506; 10.1063/1.5008478 (2017).
